# A randomized controlled study to evaluate the cost-effectiveness in sperm extraction using carbon dioxide and carbon dioxide free system in relation to intrauterine insemination pregnancy

**DOI:** 10.4103/0974-1208.63114

**Published:** 2010

**Authors:** Rajesh Bhakta, Pratap Kumar, Satish Kumar Adiga, Gurprasad Kalthur

**Affiliations:** Division of Reproductive Medicine, Department of Obstetrics and Gynecology, Kasturba Medical College, Manipal University, Manipal-576104 Karnataka, India

**Keywords:** CO_2_ incubator, intrauterine insemination, pregnancy rate, sperm preparation, water bath

## Abstract

**OBJECTIVES:**

To evaluate the effectiveness of two different systems i.e. circulating water bath and carbon dioxide (CO_2_) incubator in extracting motile sperm for IUI programme and their effect on pregnancy outcome.

**METHODS:**

The study was performed on sixty-two patients recruited for ovulation induction followed by intrauterine insemination (IUI) in University fertility clinic. The patients were randomly divided into two groups and sperm preparation was performed with either water bath or CO_2_ incubator system. The efficiency of the two systems was analyzed in relation to pregnancy outcome.

**RESULTS:**

There was no significant difference in the efficacy of water bath and CO_2_ system with respect to the quality of sperm extracted and pregnancy outcome. Although pregnancy rate was marginally higher in water bath group, it was not statistically significant.

**CONCLUSION:**

CO_2_-free system can be a cost-effective approach in IUI programme which does not compromise with pregnancy rate.

## INTRODUCTION

Sperm preparation techniques developed to separate the motile spermatozoa from seminal plasma, leukocytes, bacteria, and dead sperm plays an important role in treating infertility by intrauterine insemination (IUI) or *in vitro* fertilization (IVF). The success of IUI or IVF treatment depends upon the quality of the sperm extracted from the ejaculate.[1,2] An ideal sperm preparation method in medically assisted conception should select morphologically normal, motile sperm from the ejaculate with minimum contamination and iatrogenic damage to sperm during processing.

The most commonly followed standard swim-up technique, originally developed by Mahadevan and Baker,[3] involves centrifugal separation of spermatozoa from seminal plasma followed by incubation in media in a carbon dioxide (CO_2_) incubator maintained at 37°C and 5% CO_2_ at a pH of 7.4. Most of the commercially available sperm wash media are made up of bicarbonate-based buffer system, which requires a humidified incubator with controlled CO_2_ supply. A small-scale andrology laboratory may not be equipped with expensive instruments such as carbon dioxide incubator and its accessories. A previous study from our laboratory has proved that using a carbon dioxide free system such as water bath and media supplemented with HEPES buffer system in sperm preparation technique does not compromise with the quality of sperm extracted with respect to their yield, motility, morphology, and DNA integrity.[4] The present investigation was aimed to determine the superiority of these two methods in relation to pregnancy outcome in IUI programme.

## MATERIALS AND METHODS

### Subjects

The study was carried out in the University fertility clinic between July 2006 and April 2007 on 62 patients recruited for IUI. All women underwent basic physical examination, baseline ultrasound, tubal patency test, basal hormonal estimation, and husband semen analysis. On the day of the insemination, patients were asked to provide semen samples by masturbation after 3-5 days of ejaculatory abstinence. After liquefaction of semen, standard semen parameters (count, motility, and morphology) were evaluated according to World Health Organization (WHO) guidelines.[5]

Patients with >40 years of age, tubal factor infertility, endometriosis and cervical stenosis, duration of infertility >10 years, and abnormal endocrine profile (FSH >10 mIU/ml) were excluded from the study. Similarly, partners with azoospermia or severe oligospermia (sperm count <5 million/ml or 10 million/ml ejaculate) were also excluded. Study subjects were randomized using random number table and the semen samples were processed by swim-up technique using either water bath or CO_2_ incubator and then inseminated. A written consent was obtained from the patients enrolled for the study. The study was approved by the Institutional Ethical Committee.

### Ovulation induction and intrauterine insemination

All women were treated with 100 mg clomiphene citrate (CC) orally, from day 2 to 6 of menstrual cycle along with 75 IU human menopausal gonadotropin (HMG) intramuscularly from day 7 to 9. Follicular monitoring was done by serial transvaginal ultrasonography beginning from day 11 till the demonstration of ovulation. Human chorionic gonadotropin (HCG) 5000 IU was administered when the leading follicles were more than 18 mm. IUI was done 24 h and 48 h after the administration of the ovulation trigger.

### Sperm preparation

The sperm preparation was performed as explained elsewhere.[4] Briefly, the liquefied semen samples were washed with Earles balanced salt solution (EBSS, pH 7.4) supplemented with human serum albumin [(HSA), 0.1%] and centrifuged in a sterile tube (NUNC) to remove the seminal plasma. The motile spermatozoa were collected by overlaying the pellet with 0.4-0.5 ml of EBSS medium and incubating it for 1 h in either water bath (HAAKE, Germany) or CO_2_ incubator (HERA Cell 150, Germany). For sperm preparation using water bath system, EBSS media (pH 7.4) supplemented with HSA (0.1%) and HEPES buffer system (15 mM) was used. For sperm preparation using CO_2_ incubator system, EBSS media with bicarbonate buffer system (Sigma, USA) supplemented with 0.1% HSA was used. The motile sperm suspension collected (0.4-0.5 ml) were inseminated using IUI cannula (Gynetics Medical Products, Belgium) as per the standard protocol. The patients were instructed to lie in supine position for 15 minutes after insemination. Pregnancy was confirmed biochemically and clinically.

### Statistical analysis

Values were expressed as means ± standard error of the mean. Statistical significance was assessed using Chi-square test. *P* values of less than 0.05 were considered statistically significant.

## RESULTS

The mean age of the study subjects in CO_2_ group and water bath group was 30 ± 0.77 and 31 ± 0.73, respectively, which was not significantly different. There was no significant difference in prewash semen parameters between the two study groups [Table 1]. Furthermore, the postwash preparation after swim-up did not exhibit any significant difference between CO_2_ and water bath system with respect to the postwash count and motility. Even though the clinical pregnancy rate was marginally higher in water bath group (12.9%) compared to CO_2_ group (9.6%), the difference was statistically nonsignificant.

**Table 1:** Comparison of CO_2_ group and water bath in IUI program with respect to age, prewash, and postwash count and motility and pregnancy rates

	CO_2_ group (*N* = 31)	Water bath (*N* = 31)
Mean age (years)	30.64 ± 0.77	31.70 ± 0.73
Prewash		
Count	43.00 ± 4.5	46.32 ± 4.39
Motility	56.96 ± 3.32	53.19 ± 3.4
Postwash		
Count	16.24 ± 2.6	19.79 ± 3.7
Motility	76.67 ± 2.1	71.58 ± 3.1

## DISCUSSION

This study was aimed to evaluate two different systems in sperm processing and their efficiency in establishing pregnancy in IUI program. We have demonstrated an increase in the clinical pregnancy rate when semen sample was processed by water bath system compared to CO_2_ system. However, the differences were not statistically significant possibly due to inadequate sample size used in this study.

The cost of infertility evaluation and treatment are frequently passed directly to the patient because of limited insurance coverage and high expenses toward procurement and maintenance of the equipments. In our previous study,[4] we have demonstrated that water bath could serve as an ideal alternative to carbon dioxide incubator for the people who would like begin with only IUI program with minimum investment on equipments. Advantages of using water bath system over CO_2_ incubator are as follows: It offers a rapid and uniform temperature distribution to the sperm suspension during incubation; it is less expensive than CO_2_ incubator; and it does not compromise with the quality of the sperm prepared.

The ultimate aim of obtaining a good sperm preparation is to obtain pregnancy. The results of the present study confirm that sperm preparation method involving water bath system does not alter the pregnancy rate compared to routinely used CO_2_ system. The water bath system is not usually being used in andrology for sperm separation method most probably due to the reason that most of the commercially available sperm wash media are designed for CO_2_ system for maintaining the optimum pH, which are not suitable for maintaining pH in a closed system like water bath. However, substituting the media with a carbon dioxide independent buffer system such as HEPES buffer will help in maintaining the pH under closed system like water bath. To conclude, our study further confirms that using water bath system in IUI programme can be a cost-effective approach compared to CO_2_ system. However, this observation needs to be validated using a large number of subjects in multicentric studies.
